# Psychometric evaluation of the Mental Health Quality of Life (MHQoL) instrument in seven European countries

**DOI:** 10.1186/s12955-022-02041-6

**Published:** 2022-09-01

**Authors:** Joost J. Enzing, Frédérique C. W. van Krugten, Iryna Sabat, Sebastian Neumann-Böhme, Bert Boer, Saskia Knies, Werner B. F. Brouwer, ECOS consortium, Pedro P. Barros, Job van Exel, Jonas Schreyögg, Tom Stargardt, Aleksandra Torbica

**Affiliations:** 1 Erasmus School of Health Policy and Management, Erasmus University Rotterdam, P.O. Box 1738, 3000 DR Rotterdam, The Netherlands; 2 Zorginstituut Nederland, Diemen, The Netherlands; 3 Nova School of Business and Economics, Lisbon, Portugal; 4 Hamburg Center for Health Economics, Hamburg, Germany

**Keywords:** MHQoL, Quality of life, Mental health, Preference based measures, Psychometric evaluation

## Abstract

**Introduction:**

To make efficient use of available resources, decision-makers in healthcare may assess the costs and (health) benefits of health interventions. For interventions aimed at improving mental health capturing the full health benefits is an important challenge. The Mental Health Quality of Life (MHQoL) instrument was recently developed to meet this challenge. Evaluating the pyschometric properties of this instrument in different contexts remains important.

**Methods:**

A psychometric evaluation of the MHQoL was performed using existing international, cross-sectional data with 7155 respondents from seven European countries (Denmark, France, Germany, Italy, Portugal, The Netherlands, Portugal and the United Kingdom). Reliability was examined by calculating Cronbach’s alpha, a measure of internal consistency of the seven MHQoL dimensions, and by examining the association of the MHQoL sum scores with the MHQoL-VAS scores. Construct validity was examined by calculating Spearman’s rank correlation coefficients between the MHQoL sum scores and EQ-5D index scores, EQ-VAS scores, EQ-5D anxiety/depression dimension scores, ICECAP-A index scores and PHQ-4 sum scores.

**Results:**

The MHQoL was found to have good internal consistency for all seven countries. The MHQoL sum score and the MHQoL-VAS had a high correlation. Spearman’s rank correlation coefficients were moderate to very high for all outcomes.

**Conclusion:**

Our results, based on data gathered in seven European countries, suggest that the MHQoL shows favourable psychometrical characteristics. While further validation remains important, the MHQoL may be a useful instrument in measuring mental health-related quality of life in the Western European context.

**Supplementary Information:**

The online version contains supplementary material available at 10.1186/s12955-022-02041-6.

## Introduction

Worldwide, more than one billion people are affected by mental health problems. The burden of disease related to mental health problems is very large. For instance, the disability-adjusted life-years (DALYs) lost due to such problems represent seven percent of the total global burden of disease [1]. Additionally, mental health problems are known to have a major economic impact. This can be illustrated by the worldwide costs of lost productivity related to depression and anxiety, the two most commons mental health problems, which have been estimated to amount to US$ 1 trillion annually (1.6% of worldwide GDP) [2]. Such figures highlight the importance of effectively preventing and treating mental health problems.

However, healthcare systems have limited resources to improve the health of the populations they serve. Interventions aimed at preventing or treating mental health problems, even effective ones, in that context compete for scarce resources with interventions aimed at other diseases. Therefore, healthcare decision-makers are confronted with difficult allocation decisions. They may wish to prioritize those interventions that contribute most to the goals of the healthcare system, including using the available resources efficiently. In this context, economic evaluations are increasingly used, in which the benefits and costs of interventions (relative to some relevant comparator) are assessed in order to determine whether they are cost-effective. Such evaluations are most commonly and systematically used in relation to reimbursement decisions regarding pharmaceuticals. However, they are also increasingly applied to other types of interventions, but this broadening of the application of economic evaluations in other contexts comes with specific challenges [3]. Economic evaluations can and are also sometimes used to investigate the cost-effectiveness of mental health interventions, with the aim of informing decisions regarding their reimbursement. For mental health interventions an important challenge is capturing all relevant benefits related to these interventions. In conventional economic evaluations, health benefits are typically measured using a generic health-related quality-of-life measure, such as the EQ-5D [4] or SF-6D [5]. These instruments are prescribed in many guidelines for economic evaluations of health interventions, including the UK [6] and Dutch guidelines [7]. However, it has been questioned whether these commonly used generic health-related quality of life instruments capture all relevant quality of life domains impacted by mental health problems and interventions aimed at improving mental health [8]. This is problematic since it could lead to inaccurate estimations of health benefits related to mental health interventions, hampering well-informed decisions. Such decisions require that the quality of life impact of mental health interventions are captured accurately and completely.

In an attempt to overcome this problem, the Mental Health Quality of Life instrument (MHQoL) was developed [9]. The MHQoL is a self-report, seven-item questionnaire that captures and values dimensions relevant to the quality of life of people with mental health problems, such as self-image, independence and hope. While the instrument was based on previous research highlighting these relevant quality of life dimensions, its psychometric properties, like feasibility, reliability and validity, also need to be demonstrated. This is even more important if healthcare decisions are informed by economic evaluations using the MHQoL as outcome measure. The psychometric properties of the MHQoL so far have only been examined in the Dutch context [9]. This evaluation was performed in a sample of 110 members of the Dutch general public as well as a sample of 479 Dutch mental healthcare users. The results of this study suggested that the MHQoL is a promising instrument, demonstrating favourable psychometrical properties. However, further psychometric evaluation in different populations and contexts remains warranted. This is also true for psychometrical evaluation in an international context. Such evaluation may be considered relevant, not only to investigate the performance of translated versions of the MHQoL, but also given that cultural differences may impact how mental health problems may be experienced, evaluated and perceived [10].

The objective of this study, therefore, is to evaluate the psychometric characteristics of the MHQoL using a large dataset obtained in seven European countries. Specifically, we will investigate the reliability of the MHQoL by examining its internal consistency as well as the construct validity of the MHQoL by investigating the association of the MHQoL sum scores with other validated outcome measures.

## Methods

### Data source

For this study, we used cross-sectional data obtained in the fourth survey wave of the European COvid Survey (ECOS) project, which is described in detail elsewhere [11]. Generally, this online survey examined support for COVID-19 containment policies, including vaccinations, worries about COVID-19, and trust in different information sources. The data in the fourth wave of this survey was obtained between 5 and 16 November 2020. Respondents (n = 7115) were recruited from the general public in seven European countries (Denmark, France, Germany, Italy, Portugal, the Netherlands, and the United Kingdom) by the market research company Dynata using multisource online panels. To ensure that the sampling frame was representative of the population in each country, the company used various recruiting procedures for different subgroups of the population in each country. It used for example advertised/open recruitment, loyalty programs, affiliate networks and mobile apps [11]. Quotas based on age category, regional distribution and gender were implemented by the authors using the Qualtrics research suite to ensure and control the representativeness based on the country specific census data. Dynata ensured representativeness with regard to educational categories based on their expertise in the differences in educational degrees for each country. The authors proceeded by excluding incompletes answers and speeders (faster than 1/3 of the median time in each country), both of which were replaced by Dynata to ensure the representativeness of the sample. The resulting sample of respondents from each country (with n ~ 1000) was representative of its adult population in terms of region, gender, age group and education level.

The questionnaire was available in the seven languages of the included countries. The MHQoL had existing official versions in Dutch, English and German, which were used in this survey. For the other four countries and languages, the MHQoL instrument was translated by native speakers with a background in health economics.

Respondents completed the MHQoL instrument along with the EuroQol (EQ-5D-5L and EQ-VAS), ICECAP-A (the ICEpop CAPability measure for Adults), and PHQ-4 (Patient Health Questionnaire for Depression and Anxiety) instruments. For these instruments, official translations available from the developers of these instruments were used. Respondents also were asked to answer questions about their demographic characteristics including gender, age, relationship status, and level of education, next to COVID-related questions.

### Outcome measures

The MHQoL is an instrument intended to be used to describe and value respondents’ current mental health-related QoL [9]. In the descriptive part, respondents are asked to describe their mental health state using seven specific dimensions of mental health-related quality of life and four answering levels per dimension. The seven dimensions are: self-image, independence, mood, relationships, daily activities, physical health and hope. Levels for self-image for example range from ‘I think very positively about myself’ to ‘I think very negatively about myself’. Preference-based tariffs, allowing scores on the different levels to be converted into utility scores anchored on 0 (dead) and 1 (full mental health-related QoL), are not yet available for the MHQoL. In the absence of tariffs, the MHQoL sum score is used as an alternative, which ranges from 0 (lowest level on all seven dimensions) to 21 (highest level on all seven dimensions), with higher scores indicating better mental health-related quality of life. Next to the descriptive part, the MHQoL instrument also has a direct valuation part in which respondents are asked to rate their psychological wellbeing using a horizontal visual analogue scale (MHQoL-VAS) ranging from 0 (representing ‘worst imaginable psychological wellbeing’) to 10 (representing ‘best imaginable psychological wellbeing’).

The five-level EQ-5D (EQ-5D-5L) is a generic instrument to measure and value respondents’ current health-related QoL [4]. Within the questionnaire, respondents are asked to describe their health using five dimensions and five answering levels per dimension. These five dimensions are: Mobility, Self-Care, Usual Activities, Pain/Discomfort, and Anxiety/Depression. The five answering levels range from having no problems to having extreme problems. Using country-specific, preference-based tariffs, answer scores can be converted into utility scores, with 0 as the equivalent of the state ‘dead’ and 1 as the equivalent of the state ‘perfect health’. In addition to scoring the five dimensions, respondents are asked to rate their current overall health using a vertical visual analogue scale (EuroQol Visual Analogue Scale; EQ-VAS) ranging from 0 (‘worst imaginable health state’) to 100 (‘best imaginable health state’).

The ICE-CAP-A (ICEpop CAPability measure for Adults) is an instrument to measure and value respondents’ overall capability wellbeing and is grounded in Sen’s capability approach [12, 13]. Within the questionnaire, respondents are asked to describe their capabilities in relation to five important life domains: stability, attachment, autonomy, achievement and enjoyment. Each domain is scored using four levels, ranging from full capability to no capability. Using preference-based tariffs, answer scores can be converted to standardised index scores, ranging from 0 (no capability) to 1 (full capability). Currently, tariffs for the United Kingdom [12] and the Netherlands [14] are available.

Finally, the PHQ-4 is a four-item self-complete screening instrument that measures respondents’ likeliness of an anxiety disorder and/or depression [15]. The PHQ-4 is based on the Patient Health Questionnaire (PHQ), a more extensive instrument used by care providers to diagnose patients with mental health disorders. Dimensions are “Feeling nervous, anxious or on edge”, “Not being able to stop or control worrying”, “Feeling down, depressed or hopeless” and “Little interest or pleasure in doing things”. The PHQ-4 has four levels which range from “Not at all” to “Nearly every day”. A sum score can be calculated, which can be used to categorise the respondent’s psychological distress level as none (0–2), mild (3–5), moderate (6–8), or severe (9–12).

### Statistical analysis

Using descriptive statistics, the basic characteristics of the respondents were summarized. Mean MHQoL sum score and mean MHQoL-VAS score were compared to scores known for the Dutch general population [9]. For subgroups based on country, gender, and age group, MHQoL sum scores were calculated, also to provide a reference for future studies. In doing so, we do acknowledge and stress the exceptional situation due to the COVID-19 pandemic.

We additionally investigated the MHQoL dimension scores, comparing the youngest and oldest age groups. A lower and upper MHQoL quartile were distinguished using cut-off values (respectively < 12 and > 16) for the MHQoL sum score. We investigated the membership of these quartiles focusing on background characteristics of the respondents including income, mean, minimum and maximum values of the four other outcome measures. EQ-VAS mean scores per country were compared to population norm scores, also given the fact that the survey was conducted during the COVID-19 pandemic.

To examine the internal consistency of the MHQoL, Cronbach’s alpha of the seven dimensions was calculated, both overall and by country. Cronbach’s alpha is a coefficient that represents the extent to which items of a measure are correlated, indicating the extent to which these items measure the same construct, here mental health-related quality of life. Cronbach’s alpha is expressed as a number between 0 (no correlation) and 1 (full correlation), and a score of > 0.7 is seen as indicating good internal consistency [16]. In addition, Spearman’s rank correlation coefficients of the MHQoL sum score and the MHQoL-VAS were calculated, again overall and by country. This correlation was expected to be high and positive since both aim to measure mental health-related QoL. When interpreting results, a correlation coefficient is seen as trivial when < 0.1, as small when 0.1–0.3, as moderate when 0.3–0.5, as high when 0.5–0.7, as very high when 0.7–0.9, and as nearly perfect when > 0.9, following previous evaluation studies like Hoefman et al. [17]. Additionally, the association between the MHQoL-VAS and the MHQoL dimension scores was investigated using a linear regression model (ordinary least squares regression; OLS). A moderate to high positive correlation and a moderate to high adjusted R^2^ were expected since these dimensions levels represent mental health states which determine overall mental health-related QoL. An adjusted R^2^ of > 0.2 was expected, based on previous models which modelled EQ-VAS as the dependent variable and the levels of the EQ-5D dimensions as independent variables [18, 19].

To examine convergent validity, Spearman’s rank correlation coefficients between the MHQoL sum score and respectively: EQ-5D index score, EQ-VAS, EQ-5D anxiety/depression dimension score, ICECAP-A index score, and PHQ-4 sum score were calculated, overall and by country. The resulting coefficients inform whether these measures of theoretically interrelated constructs are correlated, which is an indication of construct validity. We expected the MHQoL sum score to have a moderate to strong positive correlation (0.3–0.7) with the EQ-5D index score, the EQ-VAS, and the ICECAP-A index score. This was based on the assumption that having a better mental health-related QoL is associated with both a better health-related QoL and a better wellbeing. It is acknowledged that these are complex associations, e.g. mental health has direct and indirect effects (e.g.by life style choices) on physical health and vice versa [20].

Furthermore, we expected the MHQoL sum score to have a strong negative correlation (0.5–0.7) with the EQ-5D anxiety/depression dimension and with the PHQ-4 sum score, since a better mental health-related QoL is strongly related to the absence of mental health problems. Tariffs to compute EQ-5D-5L index scores were obtained from the EuroQol website [21]. Tariffs to compute ICECAP-A index scores were obtained from the website of the University of Birmingham [12]. United Kingdom (UK) tariffs were used for EQ-5D-5L and ICECAP-A for all countries. This was done given that tariffs were not available for all countries. Since the current UK tariffs for EQ-5D-5L have been disputed [22] the analyses were also performed using Dutch EQ-5D-5L tariffs [23] to check whether this would influence results.

Beforehand, we did not formulate expectations regarding mean MHQoL sum scores in different countries although they are expected to differ, e.g. based on country differences in depression stigma [24].

All analyses were performed using STATA version 16.1 (StataCorp, 2019. Stata Statistical Software: Release 16. College Station, TX).

## Results

### Descriptive statistics

Table 1 shows the general characteristics of our respondents. About half of the respondents was female (52%), a third of the respondents lived alone (29%), and a majority of respondents were of working age (18–64 years; 79%). The mean MHQoL sum score was 14.1 (SD ± 3.8), which is below the MHQoL sum score reported for the Dutch general population (15.5; SD ± 2.9) [9]. The mean MHQoL-VAS was 6.6 (SD ± 2.2), which is below the mean MHQoL-VAS reported for the Dutch general population (7.5; SD ± 1.5) [9]. For the dimension scores (scale 0 to 3; higher is better), means ranged from 1.8 (Hope) to 2.2 (Physical health).

**Table 1:** Respondent characteristics (n = 7115)

	%	N
*Gender*		
Female	52.0	3699
*Age category*		
18-24	9.9	707
25-34	15.8	1125
35-44	18.9	1343
45-54	18.7	1332
55-64	16.1	1145
65+	20.6	1463
*Relationship status*		
Married/registered partnership	48.0	3416
Living together (relationship)	14.4	1027
Living alone (single)	24.0	1710
Living alone (in a relationship)	4.5	319
Widow/widower	3.0	212
Other	6.1	431
*Country*		
Germany	14.7	1043
United Kingdom	14.1	1006
Denmark	14.2	1012
The Netherlands	14.3	1020
France	14.3	1017
Portugal	14.3	1015
Italy	14.1	1002

Outcome measures	Mean (min, max)	Standard deviation
MHQoL sum score^a^	14.1 (0,21)	±3.8
MHQoL-VAS^a^	6.6 (0,10)	±2.2
EQ-VAS	72.3 (0,100)	±23.3
EQ-5D-5L UK tarrif	0.85 (-0.285,1)	±0.2
ICECAP-A UK tarrif	0.78 (-0.001,1)	±0.2
PHQ-4 sum score^b^	3.4 (0,12)	±3.3

MHQoL dimension score^a^	Mean (min, max)	Standard deviation
Self-image	1.9 (0,3)	±0.7
Independence	2.0 (0,3)	±0.8
Mood	2.2 (0,3)	±0.8
Relationships	2.1 (0,3)	±0.8
Daily activities	1.9 (0,3)	±0.8
Physical health	2.2 (0,3)	±0.8
Hope	1.8 (0,3)	±0.8

*Min* minimum value, *Max* maximum value, *MHQoL* Mental health quality of life, *EQ-VAS* EuroQol Visual Analogue Scale, *EQ-5D-5L* EuroQol 5 dimensions 5 levels, *ICECAP-A* ICEpop CAPability measure for Adults, *PHQ* Patient Health Questionnaire-4, *UK* United Kingdom

^a^Higher is better mental health-related QoL

^b^Lower is better mental health

The observed mean MHQoL sum score differed somewhat between subgroups, see Table 2. This Table also shows that the mean MHQoL sum score increased with age.

**Table 2:** Mean MHQoL scores by country, gender and age group

	Mean MHQoL sum score^a^	Standard deviation
Overall	14.1	± 3.8
*Country*		
Germany	14.5	± 3.6
United Kingdom	14.0	± 4.4
Denmark	14.7	± 3.7
the Netherlands	14.9	± 3.7
France	13.3	± 3.8
Portugal	14.1	± 3.4
Italy	13.3	± 3.8
*Gender*		
Male	14.6	± 3.7
Female	13.7	± 3.9
*Age category (years)*		
18–24	13.4	± 3.9
25–34	13.9	± 4.0
35–44	13.9	± 4.0
45–54	13.8	± 3.9
55–64	14.4	± 3.6
65+	14.9	± 3.5

^a^Minimum 0, Maximum 21. A higher MHQoL sum score means a better mental health-related QoL

Mean MHQoL sum scores, by country and age group, are presented in Table 3. These scores ranged from 12.3 in young adults (18–24 years) in the UK, to 15.9 in Danish elderly (65+ years).

**Table 3:** Mean MHQoL sum score by country and age group

	Germany	United Kingdom	Denmark	The Netherlands	France	Portugal	Italy
18–24 years	13.9	12.3	14.0	13.6	12.8	13.6	12.5
25–34 years	14.5	13.4	14.6	14.6	13.2	14.2	12.8
35–44 years	14.1	13.5	14.3	14.7	13.3	14.1	13.6
45–54 years	14.5	13.6	14.0	14.6	13.0	14.0	12.9
55–64 years	14.4	14.8	15.0	15.4	13.6	14.2	13.4
65+ years	15.0	15.4	15.9	15.7	13.8	14.8	13.9
Overall	14.5	14.0	14.7	14.9	13.3	14.1	13.3

Minimum 0, Maximum 21. Higher is better mental health-related QoL

Given the observed increase in MHQoL with age, we decomposed the MHQoL sum score into domain scores for the youngest (18–24 years) and oldest (65 + years) age groups, between which the difference was the largest (1.6 points) (see Additional file 1). The youngest group scored worse than the oldest group on all dimensions except for Physical health (+ 0.2), while especially their scores on the dimensions Mood (-0.6) and Independence (-0.5) were relatively low.

Characteristics and mean measure scores are presented in Table 4 contrasting the respondents with a high (> 16) MHQoL score and those with a low (< 12) MHQoL scores. Compared to those in the high group, respondents in the low group were younger, less often in a relationship, more often female, had lower wellbeing scores, and a lower health-related QoL.

**Table 4:** Descriptive statistics of the highest and lowest MHQoL quartiles

	High MHQoL score group^a^ (SD) (min, max)	Low MHQoL scores groupb (SD) (min, max)
Female	47%	61%
Age (mean)	50 (± 16.5) (18,90)	44 (± 15.6) (18,85)
% Household’s total monthly income measured as being able to make ends meet (fairly) easily	76%	33%
*Relationship status*		
Married/registered partnership	56%	36%
Living together (relationship)	15%	15%
Living alone (single)	19%	33%
*Age category*		
18–24	7%	14%
25–34	16%	18%
35–44	18%	20%
45–54	17%	21%
55–64	17%	13%
65+	26%	14%
*Measures (mean)*		
MHQoL sum score	18.46 (± 1.4) (17,21)	8.58 (± 2.6) (0,11)
EQ-VAS	83 (± 19.6) (0,100)	57 (± 24.7) (0,100)
EQ-5D UK tarrif	0.94 (± 0.12) (− 0.094,1)	0.70 (± 0.22) (− 0.285,1)
ICECAP-A UK tarrif	0.91 (± 0.10) (0.156,1)	0.57 (± 0.21) (− 0.001,1)
PHQ-4 sum score	1.51 (none) (± 2.5) (0,12)	6.07 (moderate) (± 03.2) (0,12)

SD standard deviation, Min minimum value, Max maximum value

^a^MHQoL sum score > 16 (n = 1849)

^b^MHQoL sum score < 12 (n = 1564)

The mean score on the EQ-VAS in our sample was 72.3 (SD ± 23.3), which represents a moderate health-related QoL. For all six countries for which EQ-VAS population norm scores were available—these were not available for Portugal—the mean EQ-VAS-score was below this norm (which ranges from 76.8 in France to 83.7 in Denmark) [25]. Overall, health-related QoL was relatively low compared to reference values.

In contrast to population norms [25], the mean EQ-5D scores in our sample did not decrease with age. For example, overall in the age group 18–24 the EQ-VAS score was 71.6 (SD ± 25.1) while it was 73.0 (SD ± 21.5), for those over 65. We also did not observe a decrease with age in mean scores for capability wellbeing (ICECAP-A scores).

### Internal consistency

The seven dimensions of the MHQoL were found to have good internal consistency, with an overall Cronbach’s alpha of 0.82. Comparable levels of internal consistency were observed for all individual countries (presented in Table 5), with alphas ranging from 0.78 for Portugal to 0.87 for the United Kingdom.

**Table 5:** Chronbach’s alpha by country

Country	Scale reliability coefficient
Germany	0.81
United Kingdom	0.87
Denmark	0.82
the Netherlands	0.84
France	0.80
Portugal	0.78
Italy	0.83
Overall	0.82

### MHQoL and MHQoL-VAS correlation

As expected, a significant positive correlation (0.65; p < 0.00) was found between MHQoL sum score and the MHQoL-VAS. For the individual countries, Spearman’s rank correlation coefficient ranged from 0.53 (Italy) to 0.67 (Portugal and the United Kingdom).

The adjusted R^2^ of the multivariate linear model (OLS; Table 6) with MHQoL-VAS as the dependent and the MHQoL dimension level scores as the independent variables was 0.43 for the total sample. For the individual countries, this ranged from 0.36 (Italy) to 0.51 (Portugal).

**Table 6:** Multivariate regression analysis of MHQoL-VAS

	MHQoL-VAS	Coefficient	95% confidence interval
*Self-image (MHQoL1)*			
	I think very positively about myself	Reference	
	I think positively about myself	− 0.10	[− 0.23, 0.03]
	I think negatively about myself	− 0.68*	[− 0.85, − 0.51]
	I think very negatively about myself	− 1.28*	[− 1.57, − 0.99]
*Independence (MHQoL2)*			
	I am very satisfied with my level of independence	Reference	
	I am satisfied with my level of independence	− 0.42*	[− 0.53, − 0.32]
	I am dissatisfied with my level of independence	− 0.58*	[− 0.72, − 0.43]
	I am very dissatisfied with my level of independence	− 0.57*	[− 0.78, − 0.36]
*Mood (MHQoL3)*			
	I do not feel anxious, gloomy, or depressed	Reference	
	I feel a little anxious, gloomy, or depressed	− 0.95*	[− 1.05, − 0.86]
	I feel anxious, gloomy, or depressed	− 1.53*	[− 1.68, − 1.38]
	I feel very anxious, gloomy, or depressed	− 2.02*	[− 2.25, − 1.78]
*Relationships (MHQoL4)*			
	I am very satisfied with my relationships	Reference	
	I am satisfied with my relationships	− 0.19*	[− 0.29, − 0.09]
	I am dissatisfied with my relationships	− 0.48*	[− 0.62, − 0.34]
	I am very dissatisfied with my relationships	− 0.53*	[− 0.75, − 0.30]
*Daily activities (MHQoL5)*			
	I am very satisfied with my daily activities	Reference	
	I am satisfied with my daily activities	− 0.24*	[− 0.35, − 0.12]
	I am dissatisfied with my daily activities	− 0.44*	[− 0.59, − 0.29]
	I am very dissatisfied with my daily activities	− 0.86*	[− 1.09, − 0.62]
*Physical health (MHQoL6)*			
	I have no physical health problems	Reference	
	I have some physical health problems	− 0.19*	[− 0.28, − 0.10]
	I have many physical health problems	− 0.49*	[-0.64, − 0.35]
	I have a great many physical health problems	− 0.85*	[− 1.08, − 0.63]
*Future (MHQoL7)*			
	I am very optimistic about my future	Reference	
	I am optimistic about my future	− 0.04	[− 0.17, 0.10]
	I am gloomy about my future	− 0.67*	[− 0.83, − 0.51]
	I am very gloomy about my future ()	− 0.95*	[− 1.17, − 0.72]
Constant		8.70*	[8.57, 8.82]
Adjusted R-squared		0.43	

*p < 0.001

MHQoL-VAS scores were most strongly associated with Mood (all levels), Self-image (‘very negative’) and Future (‘very gloomy about’). This might suggest that these will also receive the most weight in future MHQoL tariffs. Within each dimension lower levels were associated with lower MHQoL-VAS scores. The only exception were the scores associated with the third and fourth level for the domain Independence, which were almost identical. All but two coefficients were statistically significant.

### Convergent validity

To examine convergent validity, an element of construct validity, Spearman’s rank correlation coefficients were calculated for MHQoL sum scores with other relevant outcome measures. The resulting rank correlations are presented in Table 7. As expected, strong positive correlations were observed between MHQoL sum score and EQ-VAS, EQ-5D index scores and ICECAP-A index scores. The additional analysis using Dutch instead of UK EQ-5D tariffs resulted in comparable correlation coefficients (not presented). Furthermore, expected strong negative correlations were observed with the EQ-5D dimension anxiety and depression and with the PHQ4 score.

**Table 7:** Spearman’s rank correlation coefficients

Examined rank correlation of MHQoL sum score	Spearman’s rho
*EQ-5D-5L dimension anxiety and depression (higher is worse)*	
Overall	− 0.63*
Germany	− 0.60*
United Kingdom	− 0.71*
Denmark	− 0.61*
the Netherlands	− 0.66*
France	− 0.63*
Portugal	− 0.59*
Italy	− 0.60*
*EQ-VAS (higher is better QoL)*	
Overall	0.49*
Germany	0.50*
United Kingdom	0.58*
Denmark	0.53*
the Netherlands	0.53*
France	0.45*
Portugal	0.43*
Italy	0.46*
*EQ-5D-5L index score (UK tariffs) (higher is better QoL)*	
Overall	0.56*
Germany	0.57*
United Kingdom	0.62*
Denmark	0.56*
the Netherlands	0.62*
France	0.56*
Portugal	0.58*
Italy	0.54*
*PHQ4 sum score (higher is worse mental health)*	
Overall	− 0.60*
Germany	− 0.54*
United Kingdom	− 0.73*
Denmark	− 0.45*
the Netherlands	− 0.65*
France	− 0.58*
Portugal	− 0.57*
Italy	− 0.60*
*ICECAP-A index score (UK tariffs) (higher is better wellbeing)*	
Overall	0.68*
Germany	0.64*
United Kingdom	0.75*
Denmark	0.70*
the Netherlands	0.71*
France	0.64*
Portugal	0.67*
Italy	0.64*

*p < 0.001

## Discussion

In this paper, we presented a psychometric evaluation of the MHQoL questionnaire in seven European countries. This study, therefore, represents one of the first psychometric evaluations of the MHQoL and the first international one to our knowledge. Importantly, this builds on the evidence of the performance of the MHQoL as a reliable and valid measure of mental health-related QoL across Western European countries.

Specifically, we examined reliability by investigating internal consistency and construct validity by investigating convergent validity, using existing survey data obtained through the ECOS survey, which was conducted in seven European countries. Overall as well as for the separate counties, we found good internal consistency between the dimensions. Additionally, the MHQoL-VAS score, in general, was significantly associated with the MHQoL sum scores and domain scores in the expected way. Convergent validity was investigated by investigating the correlations between the MHQoL instrument with the EQ-5D, ICECAP-A as well as specific mental health instruments and showed favourable results. Overall our results suggest that in the investigated countries, the MHQoL appears to be a psychometrically sound measure of quality of life in people with mental health problems, which highlights it is a promising instrument to use and validate further.

The importance in doing so may be emphasised by the fact that sound and concise instruments, capable of adequately capturing important life domains impacted by mental health problems, are currently lacking [26]. Instruments like the MHQoL, therefore, can facilitate broadening the application of HTA to the field of mental health in a way that the benefits of mental health interventions are indeed captured. This may ultimately lead to better (informed) decision-making and, therefore, a more efficient and equitable allocation of resources.

## Limitations of the study

The main strengths of our study are its large sample size, the representativeness and international character of its sample, the inclusion of several relevant outcome measures, and the evaluation of multiple psychometric properties. Besides these strengths, several limitations need to be acknowledged. First, the psychometric evaluations performed were not without limitations. Given the nature of the ECOS survey, we did not have any information on clinical diagnosis of respondents. Consequently, we could not evaluate whether MHQoL scores were associated with the absence, presence or severity of mental health problems as determined by clinical professionals. Such an evaluation would have been a valuable and relevant addition to the results presented here, which relied on self-reported outcome measures. Furthermore, since the MHQoL was only included in one of the ECOS survey waves, we were not able to perform a test–retest validation. This would have allowed further evaluation of the reliability (consistency in measurement) and responsiveness (ability to capture changes in mental health) of the MHQoL. In addition, in the absence of MHQoL tariffs, we based our analyses on the sum scores and VAS scores of the MHQoL, while basing them on utility scores would have been interesting and insightful as well.

Second, the data were collected using an online survey, which may have caused some selection bias in respondents and may be associated with lesser engagement with the survey by respondents. Moreover, and very important to stress, the data were collected during the COVID-19-crisis. This situation, the threat or experience of falling ill as well as the strong measures imposed in most countries to mitigate the outbreak, may have affected mental health of the population. The fact that the survey focused on COVID-related aspects (including risks, worries and government intervention) may have increased the awareness of negative consequences and, therefore, negative feelings. The QoL and wellbeing scores observed in our study were, on average, indeed lower than previously observed. The age-profile for mental health, with younger people scoring lower than expected and lower than older people in all domains except for physical health, may also be related to this context, if the mental health of young adults on average would be affected more due to COVID (measures) than that of the old (e.g. by affecting normal activities and social life more in the young). As a competing explanation, the online form of the survey may have caused a selection bias in the group of older people towards those with a higher level of mental health. While this may not have affected the results in relation to our research question on the validity of the MHQoL, it is important to emphasize this when using the here presented MHQoL scores as a reference in future studies.

As a third limitation, the MHQoL instrument was translated from Dutch to four other languages by native speakers with a background in health economics. A more extensive translation procedure, as was done for English and German, using forward–backward techniques, would have been superior.

## Future perspective

Given the findings presented here, the MHQoL appears to be a valid and reliable measure of mental health-related QoL in the Western European context. More research, confirming these findings, and expanding the investigation to other aspects of feasibility, reliability and validity, is required in order to gain a fuller understanding of the psychometric properties of the MHQoL, also in different groups (including people with known mental health problems). If the current positive findings are confirmed, the MHQoL can be used in different settings to monitor or evaluate (changes in) mental health-related quality of life. This comprehensive measure may also be a valuable addition to disease-specific clinical outcomes in clinical decision making e.g. to inform patients on the expected impact of an intervention on their quality of life. It could also be applied in economic evaluations, informing decision-makers about the full costs and benefits of mental health interventions. It needs noting that in that context, using the MHQoL rather than generic HR-QoL measures, has the advantage of being more comprehensive in terms of relevant health domains covered but comes at the price of limiting comparability between outcomes. This may be less problematic for decisions within the mental health domain. Moreover, if adequate thresholds would be established for the MHQoL (like for generic HRQoL), decision-making would be more straightforward. However, given that in several Western European countries (with Germany as an exception) [27] the EQ-5D currently is prescribed to be used in economic evaluations (also within the mental health field), for these countries administering the MHQoL instrument *alongside* the EQ-5D is recommendable in the context of economic evaluations. This will also offer the opportunity to further investigate the relationship between the EQ-5D and MHQoL instruments.

The use of the MHQoL in economic evaluations would require having valid tariffs that transform a MHQoL state as described on the descriptive system into a utility score (typically anchored on the states ‘dead’ (0) and ‘perfect health’ (1). Such tariffs are expected to be developed soon (for the Netherlands).

## Conclusion

Our results suggest the MHQoL is a psychometrically sound measure of mental health-related quality of life in the Western European context. While more research remains necessary, this makes the MHQoL instrument interesting to be used in (economic) evaluations of mental health interventions, as it more comprehensively captures their benefits.

## Supplementary Information

**Additional file 1**. Dimension scores per age category.

## Data Availability

The MHQoL instruments are available from the website https://www.imta.nl/mhqol. Copyright holder of the MHQoL is the Erasmus School of Health Policy & Management, Erasmus University Rotterdam, Rotterdam, The Netherlands.

## References

[1] Rehm J, Shield KD (2019). Global burden of disease and the impact of mental and addictive disorders. Curr Psychiatry Rep.

[2] Chisholm D, Sweeny K, Sheehan P (2016). Scaling-up treatment of depression and anxiety: a global return on investment analysis. Lancet Psychiatry.

[3] Enzing JJ, Knies S, Boer B (2021). Broadening the application of health technology assessment in the Netherlands: a worthwhile destination but not an easy ride?. Health Econ Policy Law.

[4] Herdman M, Gudex C, Lloyd A (2011). Development and preliminary testing of the new five-level version of EQ-5D (EQ-5D-5L). Qual Life Res.

[5] Brazier J, Roberts J, Deverill M (2002). The estimation of a preference-based measure of health from the SF-36. J Health Econ.

[6] NICE (2013). Guide to the methods of technology appraisal 2013.

[7] ZIN (2015). Richtlijn voor het uitvoeren van economische evaluaties in de gezondheidszorg.

[8] Brazier J (2010). Is the EQ-5D fit for purpose in mental health?. Br J Psychiatry.

[9] van Krugten FCW, Busschbach JJV, Versteegh MM (2022). The Mental Health Quality of Life Questionnaire (MHQoL): development and first psychometric evaluation of a new measure to assess quality of life in people with mental health problems. Qual Life Res.

[10] Kleinman A. Rethinking Psychiatry: from cultural category to personal experience. New York: Free Press (Macmillan Publishing Co.), 1988.

[11] Sabat I, Neuman-Bohme S, Varghese NE (2020). United but divided: Policy responses and people’s perceptions in the EU during the COVID-19 outbreak. Health Policy.

[12] Flynn TN, Huynh E, Peters TJ (2015). Scoring the Icecap-a capability instrument. Estimation of a UK general population tariff. Health Econ.

[13] Al-Janabi H, Flynn TN, Coast J (2012). Development of a self-report measure of capability wellbeing for adults: the ICECAP-A. Qual Life Res.

[14] Rohrbach PJ, Dingemans AE, Essers BA (2021). The ICECAP-A instrument for capabilities: assessment of construct validity and test-retest reliability in a general Dutch population. Qual Life Res.

[15] Kroenke K, Spitzer RL, Williams JB (2009). An ultra-brief screening scale for anxiety and depression: the PHQ-4. Psychosomatics.

[16] Tavakol M, Dennick R (2011). Making sense of Cronbach’s alpha. Int J Med Educ.

[17] Hoefman RJ, van Exel J, Brouwer WB (2013). Measuring the impact of caregiving on informal carers: a construct validation study of the CarerQol instrument. Health Qual Life Outcomes.

[18] Feng Y, Parkin D, Devlin NJ (2014). Assessing the performance of the EQ-VAS in the NHS PROMs programme. Qual Life Res.

[19] Devlin N, Parkin D, Janssen B (2020). Analysis of EQ VAS data.

[20] Ohrnberger J, Fichera E, Sutton M (2017). The relationship between physical and mental health: a mediation analysis. Soc Sci Med.

[21] Devlin NJ, Shah KK, Feng Y (2018). Valuing health-related quality of life: an EQ-5D-5L value set for England. Health Econ.

[22] Wailoo A, Alava MH, Pudney S (2021). An international comparison of EQ-5D-5L and EQ-5D-3L for use in cost-effectiveness analysis. Value Health.

[23] Mv M, Mv K, Maae S (2016). Dutch tariff for the five-level version of EQ-5D. Value Health.

[24] Coppens E, Van Audenhove C, Scheerder G (2013). Public attitudes toward depression and help-seeking in four European countries baseline survey prior to the OSPI-Europe intervention. J Affect Disord.

[25] Janssen B, Szende A, Szende A, Janssen B, Cabases J (2014). Population norms for the EQ-5D. Population norms for the EQ-5D.

[26] van Krugten FCW, Feskens K, Busschbach JJV (2021). Instruments to assess quality of life in people with mental health problems: a systematic review and dimension analysis of generic, domain- and disease-specific instruments. Health Qual Life Outcomes.

[27] Angelis A, Lange A, Kanavos P (2018). Using health technology assessment to assess the value of new medicines: results of a systematic review and expert consultation across eight European countries. Eur J Health Econ.

