# Supplementary material for: Psychometric evaluation of the Mental Health Quality of Life (MHQoL) instrument in seven European countries
Source: Health Qual Life Outcomes. 2022 Sep 1;20:129. doi: 10.1186/s12955-022-02041-6 (PMC9434504; doi:10.1186/s12955-022-02041-6)
Supplement: Supplementary file 1 — Additional file 1. Dimension scores per age category. [file 12955_2022_2041_MOESM1_ESM.docx]

**Additional file 1**

**Psychometric evaluation of the Mental Health Quality of Life (MHQoL) instrument in seven European countries**

*Joost J. Enzing^1 2^, MSc, Frédérique C.W. van Krugten^1^, PhD, Iryna Sabat^3 4^, MSc, Sebastian Neumann-B*ö*hme^4 1^, MSc,* Bert Boer^1^, PhD, *Saskia Knies^1 2^, PhD, Werner B.F. Brouwer^1^, PhD, on behalf of the ECOS consortium^[[1]](#footnote-1)^*

*1 Erasmus School of Health Policy & Management, Erasmus University Rotterdam, Rotterdam, The Netherlands*

*2 Zorginstituut Nederland, Diemen, The Netherlands*

*3 Nova School of Business and Economics, Lisbon, Portugal*

*4 Hamburg Center for Health Economics, Hamburg, Germany*

**Corresponding author**

Joost J. Enzing, Erasmus School of Health Policy & Management, P.O. Box 1738, 3000 DR Rotterdam, The Netherlands. Email: enzing@eshpm.eur.nl Phone: +31 (0)6 153 292 61

**Table S1. Dimension scores per age category**

| **Self-image (MHQoL1)** |  |  |  |  |  |
| --- | --- | --- | --- | --- | --- |
|  | **Age category** | **Mean** | **95% CI** | | |
|  | 18-24 | 1.81 | 1.76 | **-** | 1.87 |
|  | 25-34 | 1.87 | 1.82 | **-** | 1.91 |
|  | 35-44 | 1.89 | 1.86 | **-** | 1.93 |
|  | 45-54 | 1.87 | 1.83 | **-** | 1.91 |
|  | 55-64 | 1.98 | 1.95 | **-** | 2.02 |
|  | 65+ | 2.01 | 1.98 | **-** | 2.04 |
|  |  |  |  |  |  |
| **Independence (MHQoL2)** |  |  |  |  |  |
|  | 18-24 | 1.75 | 1.69 | **-** | 1.82 |
|  | 25-34 | 1.90 | 1.85 | **-** | 1.95 |
|  | 35-44 | 1.98 | 1.87 | **-** | 1.96 |
|  | 45-54 | 1.93 | 1.89 | **-** | 1.98 |
|  | 55-64 | 2.08 | 2.03 | **-** | 2.12 |
|  | 65+ | 2.19 | 2.15 | **-** | 2.23 |
|  |  |  |  |  |  |
| **Mood (MHQoL3)** |  |  |  |  |  |
|  | 18-24 | 1.85 | 1.78 | **-** | 1.92 |
|  | 25-34 | 2.08 | 2.03 | **-** | 2.13 |
|  | 35-44 | 2.14 | 2.10 | **-** | 2.19 |
|  | 45-54 | 2.18 | 2.13 | **-** | 2.22 |
|  | 55-64 | 2.33 | 2.28 | **-** | 2.37 |
|  | 65+ | 2.48 | 2.45 | **-** | 2.52 |
|  |  |  |  |  |  |
| **Relationships (MHQoL4)** |  |  |  |  |  |
|  | 18-24 | 1.97 | 1.90 | **-** | 2.03 |
|  | 25-34 | 2.10 | 2.05 | **-** | 2.15 |
|  | 35-44 | 2.07 | 2.03 | **-** | 2.12 |
|  | 45-54 | 2.11 | 2.07 | **-** | 2.16 |
|  | 55-64 | 2.21 | 2.17 | **-** | 2.26 |
|  | 65+ | 2.28 | 2.24 | **-** | 2.32 |
|  |  |  |  |  |  |
| **Daily activities (MHQoL5)** |  |  |  |  |  |
|  | 18-24 | 1.75 | 1.69 | **-** | 1.82 |
|  | 25-34 | 1.85 | 1.81 | **-** | 1.90 |
|  | 35-44 | 1.83 | 1.79 | **-** | 1.87 |
|  | 45-54 | 1.87 | 1.83 | **-** | 1.91 |
|  | 55-64 | 1.97 | 1.93 | **-** | 2.02 |
|  | 65+ | 2.04 | 2.00 | **-** | 2.08 |
|  |  |  |  |  |  |
| **Physical health (MHQoL6)** |  |  |  |  |  |
|  | 18-24 | 2.33 | 2.27 | **-** | 2.39 |
|  | 25-34 | 2.33 | 2.28 | **-** | 2.37 |
|  | 35-44 | 2.30 | 2.26 | **-** | 2.34 |
|  | 45-54 | 2.19 | 2.14 | **-** | 2.23 |
|  | 55-64 | 2.11 | 2.07 | **-** | 2.16 |
|  | 65+ | 2.11 | 2.08 | **-** | 2.15 |
|  |  |  |  |  |  |
| **Future (MHQoL7)** |  |  |  |  |  |
|  | 18-24 | 1.79 | 1.73 | **-** | 1.86 |
|  | 25-34 | 1.78 | 1.73 | **-** | 1.83 |
|  | 35-44 | 1.79 | 1.74 | **-** | 1.83 |
|  | 45-54 | 1.66 | 1.62 | **-** | 1.70 |
|  | 55-64 | 1.73 | 1.69 | **-** | 1.78 |
|  | 65+ | 1.81 | 1.77 | **-** | 1.84 |

1. ECOS consortium: Iryna Sabat, Sebastian Neumann-Böhme, Pedro P. Barros, Werner Brouwer, Job van Exel, Jonas Schreyögg, Tom Stargardt & Aleksandra Torbica [↑](#footnote-ref-1)
